# The Antitumor Effect and Mechanism of Total Flavonoids From Coreopsis Tinctoria Nutt (Snow Chrysanthemum) on Lung Cancer Using Network Pharmacology and Molecular Docking

**DOI:** 10.3389/fphar.2022.761785

**Published:** 2022-03-08

**Authors:** Yilimire Wufuer, Xu Yang, Luyuan Guo, Kasimujing Aximujiang, Li Zhong, Kurexi Yunusi, Guixia Wu

**Affiliations:** ^1^ School of Basic Medical Science, Xinjiang Medical University, Urumqi, China; ^2^ Department of Obstetrics and Gynecology, The Fifth Affiliated People’s Hospital of Chengdu University of Traditional Chinese Medicine, Chengdu, China; ^3^ Uygur Medical College, Xinjiang Medical University, Urumqi, China

**Keywords:** network pharmacology, Coreopsis tinctoria Nutt, apoptosis, lung cancer, active components

## Abstract

Coreopsis tinctoria Nutt (C. tinctoria), also known as Snow Chrysanthemum, is rich in polyphenols and flavonoids. It has important pharmacological effects such as lowering blood lipids, regulating blood glucose, and anti-tumor effect. However, its anti-tumor mechanism has not yet been investigated thoroughly. This study aimed to explore the anti-tumor effect of total flavonoids extracted from C. tinctoria (CTFs) on lung cancer and the possible mechanism. The components of CTFs were analyzed using Ultra-high-performance liquid chromatography-tandem mass spectrometry (UHPLC-MS/MS). The active components of CTFs were screened according to oral bioavailability (OB) and drug-likeness (DL). Totally, 68 components of CTFs were identified and 23 active components were screened. Network pharmacological analysis on the active components identified 288 potential targets associated with lung cancer. After protein-protein interaction (PPI) network topology analysis, 17 key protein targets including Akt1, MAPK1, TP53, Bcl-2, Caspase-3, Bax, GSK3B and CCND1 were screened. The molecular docking results showed that the active components of CTFs had good binding activity with key targets. GO and KEGG analysis of candidate targets found that the main enrichment was in PI3K/Akt-mediated intrinsic apoptotic pathways. Finally, according to the results of network pharmacology, the potential molecular mechanism of CTFs intervention in lung cancer was validated experimentally *in vitro* and *in vivo*. The experimental validation results demonstrated that the antitumor activity of CTFs on lung cancer may be related to inhibiting the PI3K-Akt signaling pathway and activating the mitochondrial-mediated apoptosis pathway.

## Introduction

At present, lung cancer is one of the most prevalent malignancies worldwide. It is the common cause of cancer-associated mortality, and its 5-year survival rate is less than 15% (Hirsch et al., 2017; Ma et al., 2018). Among all lung cancer cases, approximately 85% of patients have non-small cell lung cancer (NSCLC) (Herbst et al., 2018). The current strategies, such as surgery, radiotherapy, and chemotherapy, alone or in combination, remain palliative or unsatisfactory due to tumor metastasis or recurrence after surgery/radio-chemo-therapy, serious adverse reactions, and drug resistance (Chang, 2016; Colapietro et al., 2019).

Cancer chemoprevention involves the use of synthetic or natural agents to inhibit, retard, or reverse the process of carcinogenesis (Cragg and Pezzuto, 2016). Epidemiological and pre-clinical data suggest that multiple bioactive phytochemicals possess chemopreventive properties (Kotecha et al., 2016). Presently, natural compounds in plants, spices, fruits, and vegetables such as taxol, vinblastine, and camptothecin, have attracted considerable attention as potential candidates for anti-cancer agents (Dong et al., 2019; Fontana et al., 2020). These phytochemicals can affect proliferation, apoptosis, invasion, and migration of cancer cells and modulate multiple signaling pathways and networks that are often disrupted in tumor initiation, proliferation, and propagation (Kotecha et al., 2016). Moreover, they have the advantages of low toxicity and low cost (Colapietro et al., 2019).

Coreopsis tinctoria Nutt, native to North America, is now widely distributed worldwide (Yang et al., 2016). In China, it is mainly distributed in southern Xinjiang, especially the Karakorum and Kunlun Mountains at an altitude of 3,000 m (Begmatov et al., 2020). In North American and Portugal, *C. tinctoria* has been used to treat several diseases including diabetes, diarrhea, and internal pains (Du et al., 2018). In Traditional Chinese Medicine (TCM), it has been used to treat cardiovascular diseases, such as hypertension and hyperlipidemia (Begmatov et al., 2020). Modern pharmacological studies have confirmed that phytochemicals of *C. tinctoria* mainly contain flavonoids, phenolic acids, and terpenoids (Yang et al., 2016). The predominant flavonoids have been reported to have hypoglycemic, hypolipidemic, antioxidant, and antitumor activities (Ren et al., 2018; Zhang et al., 2019; Jing et al., 2015). However, the underlying molecular mechanisms and interactive protein targets remain unclear.

The complexity of multiple components, multiple targets, and synergistic interactions of TCM makes it difficult to carry out in-depth study. Along with the rapid development of systems biology, multi-directional pharmacology and bioinformatics, network pharmacology based on big databases provides new approaches and perspectives for the study of TCM (Huang et al., 2017). It has become a useful tool to predicte and identify the herbal targets and potential mechanisms (Li et al., 2021) through constructing “drug-target-disease” multi-level interaction network. Network pharmacology has been widely applied in the mechanism study of TCM for the treatment of complex diseases, such as cancer, cardiovascular disorders, and diabetes.

**FIGURE 1:**
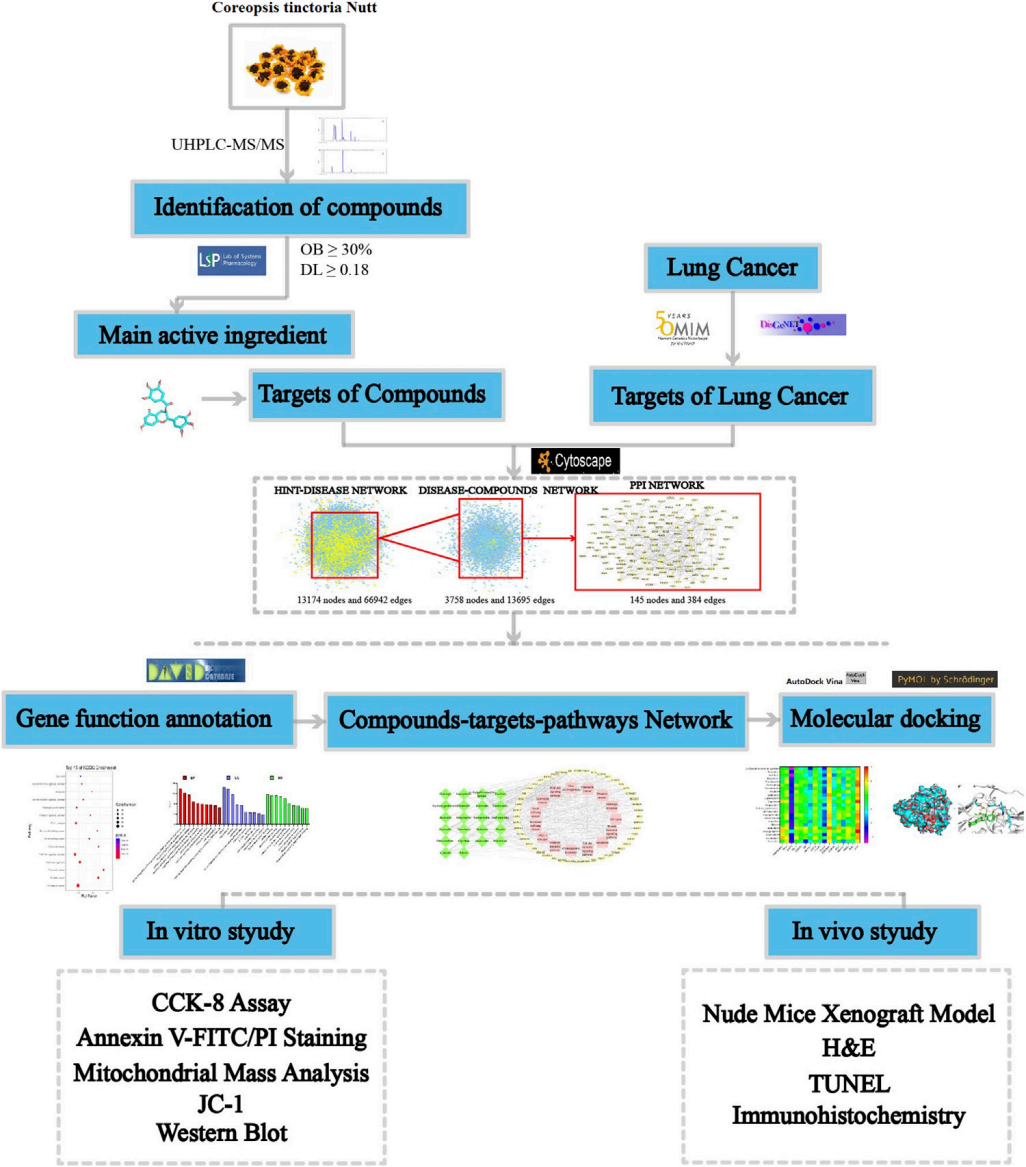
The CTFs of the network pharmacological analysis approach.

In the present study, ultra-high-performance liquid chromatography-tandem mass spectrometry (UHPLC-MS/MS) was used to identify components of total flavonoids extracted from *C. tinctoria* (CTFs). Based on network pharmacology, we performed cytotoxicity screening, cellular and molecular biology validation to identify the antitumor effective targets and mechanism of CTFs. The study workflow is shown in Figure 1.

## Materials and Methods

### Reagents

The *C. tinctoria* was from the research base of the Uyghur Autonomous Region Institute of Medicinal Plants (Xinjiang, China). Human lung carcinoma (A549 and NCI-H292) cells and BEAS-2B were purchased from the Cell Bank of the Chinese Academy of Sciences (Shanghai, China). RPMI-1640 medium and fetal bovine serum (FBS) was from Gibco (United States). Penicillin/streptomycin, and dimethyl sulfoxide (DMSO) were purchased from Sigma (Missouri, United States). Cell Counting Kit-8 (CCK-8) and Annexin V-FITC/PI apoptosis detection kits were from BD Biosciences (United States). HPLC grade methanol and acetonitrile were purchased from Merck (Darmstadt, Germany). The antibodies of Caspase-3, Bax, and Bcl-2 were all obtained from Abcam (United States); those of p-PI3K, PI3K, p-Akt, and Akt were from Affinity Biosciences (Cincinnati, United States); and, that of β-actin was from Proteintech Group (Wuhan, China). An enhanced chemiluminescence (ECL) kit was purchased from PerkinElmer (Waltham, United States).

### Extraction of Total Flavonoids

The total flavonoids with a purity of 80% (w/w) were extracted by evaporator method and purified by Sephadex LH-20 column. In brief, the *C. tinctoria* was dried at 60°C, made into powders, and filtered through a 60-mesh sieve. The dried powder was incubated in 70% ethanol (1:10, w/v) for 24 h, filtered, and extracted twice in a vacuum rotary evaporator at 70°C, each time for 2 h. The extracted solutions were combined and concentrated to yield a dried residue. The residue was then subjected to HPD-100A resin and eluted with H_2_O, 20, 40, and 65% ethanol, successively. The fraction eluted with 40% ethanol was subjected to Sephadex LH-20 column and eluted with 10–90% methanol for purification of total flavonoids.

### Cell Culture

A549, H292, and BEAS-2B cells were cultured in RPMI-1640 medium supplemented with 10% FBS and 1% penicillin/streptomycin in a humidified atmosphere of 95% air and 5% CO_2_ at 37°C. The medium was subsequently replaced at 2-day intervals.

### CCK-8 Assay

The CCK-8 assay was used to determine the effect of CTFs on the cell viability of cells. Briefly, the cells were seeded onto 96-well plates at 1 × 10^4^ cells/well for 24 h and then incubated with various concentrations of CTFs (25, 50, 100, 200, 400, 600, 800 or 1,000 µg/ml) for 24, 48, and 72 h at 37°C. The cells treated by DMSO were used as negative control. Following incubation, CCK-8 was added to each well, followed by incubation for 2 h at 37°C. The optical density (OD) at 490 nm was measured using a microtiter plate reader. Cell viability (%) = OD (experimental group)/OD (control group) × 100%. Each experiment was performed in triplicate.

### Chemical Profile Analysis by UHPLC-MS/MS

UHPLC-MS/MS was used to determine the chemical profile of CTFs. The separation was carried out on Waters Acquity UPLC, BEH C18 (1.7 μm, 2.1*150 mm) at a flow rate of 0.3 ml/min. The mobile phase consisted of a combination of A (0.1% formic acid in water, v/v) and B (acetonitrile). AB Sciex QTrap 6500 + mass spectrometer was applied for assay development. Typical ion source parameters were: IonSpray Voltage: +5000/−4500 V, Curtain Gas: 35 psi, Temperature: 50°C, Ion Source Gas 1: 55 psi, Ion Source Gas 2:60 psi.

### Prediction of Drug Targets for CTFs

To evaluate the pharmacokinetic properties of CTFs, all compounds were analyzed using the TCMSP database (http://lsp.nwu.edu.cn/). The active components of CTFs were screened according to the criteria of oral bioavailability (OB) ≥ 30% and drug-likeness (DL) ≥ 0.18. Then, the targets of the active components were predicted by the TCMSP platform and ETCM database (http://www.tcmip.cn/ETCM/index.php). The STRING database (https://string-db.org/) and the “Homo sapiens” species settings were used to identify and standardize the target proteins of the active components of CTFs.

### Construction and Analysis of Target Network

The lung cancer-related protein targets were screened using the following two databases: OMIM (http://www.ncbi.nlm.nih.gov/omim/) and the DisGeNET (http://www.disgenet.org/). We screened the predicted targets using “lung cancer” as the keyword. All high-quality binary protein-protein interactions of human species were downloaded from the high-quality proteomics database HINT (High-quality INTeractomes) to construct a human genome-wide protein-protein interaction network (PPI). All disease targets of lung cancer were mapped to the HINT background network. After extracting the lung cancer network, the CTFs targets were mapped to the lung cancer network. All target nodes and the edges between them were collected, and only the largest connected branch was retained to obtain the target network. Through network topology analysis, potential targets were obtained.

### GO and KEGG Pathway Enrichment Analysis

Potential targets were imported into the DAVID 6.8 database (https://david.ncifcrf.gov/) to perform GO enrichment analysis and KEGG pathway enrichment analysis. “OFFICE_GENE_SYMBOL” and “Homo sapiens” were selected. The top 10 enriched GO entries and top 15 enriched KEGG pathways that met the criteria of *p* < 0.01 were selected, and uploaded to the OmicShare (http://www.omicshare.com/tools/) cloud platform for data visualization, where the size of the bubble represents the number of genes in the pathway, and the color represents the significance of enrichment.

### Construction of Core Component-Target-Pathway Network.

The integrated network of component-target-pathway was constructed using Cytoscape 3.6.0 to identify the relationships of protein targets with each compound and the involved pathways and diseases. The topology characteristics of the network were evaluated.

### Molecular Docking of Active Ingredient-Key Target

To further verify the reliability of the target prediction results, the key targets with degrees greater than the average degree in the “component-target-pathway” network were selected and Auto dock vina (Trott and Olson, 2010). was used for molecular docking to verify the binding activity of key target with components.

### Apoptosis Assay With Annexin V-FITC/PI Staining

A549 and H292 cells were plated into 6-well plates (1 × 10^6^ cells/well) and treated with different concentrations of CTFs (25, 50, 100, and 200 µg/ml) for 24 h. The cells were collected and stained with Annexin V-FITC/PI according to the manufacturer’s protocol for 15 min in the dark. Apoptotic cell ratio was detected using a flow cytometer. Each experiment was done in triplicates.

### Mitochondrial Mass Analysis

Mitochondrial mass was observed by the fluorescent probe Mito-tracker Red CMXRos (Beyotime, Shanghai, China). Cells were incubated with different concentrations of CTFs (50, 100, and 200 µg/ml) for 24 h, and then treated with the Mito-tracker Red CMXRos for 30 min at 37°C in the dark. The results of mitochondrial staining was observed by confocal fluorescence microscopy (Leica Microsystems, Heidelberg, Germany).

### Mitochondrial Membrane Potential Measurement

MMP was detected by the JC-1 (Beyotime, Shanghai, China). Cells were incubated with different concentrations of CTFs (50, 100, 200 µg/ml) for 24 h. Then, cells were stained with JC-1 at 37°C in the dark for 20 min. After washing 2 times with PBS, JC-1 aggregates in cells were analyzed by confocal microscope. The ratio of red/green fluorescence intensity was calculated with the ImageJ software.

### Western Blot

After treatment with CTFs (0, 50, 100, and 200 µg/ml) for 24 h, A549 cells were collected and lysed with RIPA lysis buffer (containing a 1% protease inhibitor cocktail). The protein concentration was measured with the BCA Protein Assay Kit. The protein samples were separated by 10% SDS-PAGE and transferred onto PVDF membranes (Millipore, Bedford, MA, United States). After blocking with 5% (v/v) non-fat milk for 2 h, membranes were incubated with primary antibodies overnight at 4°C. The primary antibodies included anti-caspase 3 (1:2,000), anti-Bcl-2 (1:1,000), anti-Bax (1:1,000), anti-p-PI3K (1:1,000), anti-PI3K (1:1,000), anti-p-Akt (1:1,000), anti-Akt (1:1,000), and anti-β-actin (1:500). The membranes were incubated with a horseradish peroxidase-conjugated secondary antibody for 2 h at room temperature. The protein bands were visualized using ECL detection reagents (Bio-Rad, United States) and the band density was evaluated using ImageJ software.

### Nude Mice Xenograft Model

Nude mice (BALB/c null; 6-week-old) were purchased from Beijing Vital River Laboratory Animal Technology Co., Ltd. (China). The Institutional Animal Care and Use Committee at Xinjiang Medical University approved all animal experiments in this study. Nude mice were given sterile food and water and kept in pathogen-free conditions. A549 cells (1 × 10^7^) were injected subcutaneously into the left flanks of mice. Tumor length (a) and width (b) were measured using Vernier calipers every 3 days. Tumor volume was calculated using the formula: V (mm^3^) = 1/2*ab^2^. Following cell injection for 14 days, mice were randomly divided into four groups (*n* = 6): model group; low dose CTFs group (50 mg/kg); medium dose CTFs group (100 mg/kg); high dose CTFs group (200 mg/kg) and positive control group (5-fluorouracil, 50 mg/kg). Drugs were dissolved in carboxymethyl cellulose sodium salt and administered by gavage. The body weight and tumor volume of mice were measured every 3 days until the 24th day. Then, mice were sacrificed, and tumors were collected and stored at −80°C.

### Hematoxylin-Eosin (H&E)

Nude mouse tumor tissues were fixed with 10% neutral formaldehyde, routinely dehydrated, embedded in paraffin, sectioned, and stained with HE. The morphological changes of the tumor tissues in each group were observed under an optical microscope.

### TUNEL Assay Analysis of Cell Apoptosis

Cell apoptosis in mice tumor tissues was examined using TUNEL assay (Beyotime Biotech, Wuxi, China) according to the manufacturer’s instructions. The sections were incubated with 20 μL of TUNEL reaction mixture for 30 min in a humid chamber in the dark, and then the sections were washed three times (5 min each) with TBS. Then, 50 μL streptavidin-HRP solution (1:100) was added room temperature, followed by three washes with TBS (5 min each) and DAPI redyeing. The slices were mounted and observed under fluorescence microscope.

### Immunohistochemistry

The xylene deparaffinized and hydrated tissue sections were incubated with 3% H_2_O_2_ to block endogenous peroxidase for 20 min at room temperature. After washing with PBS (3 min × 3 times), normal goat serum was added for blocking at 37°C for 30 min. Primary antibodies of Bax, Bcl-2, p-PI3K, and p-Akt were then added for incubation overnight at 4°C. After washing again, the secondary antibody of polyperoxidase-anti-Rabbit IgG was added and incubated at 37°C for 30 min. DAB was used for color development. The counterstaining with hematoxylin was performed. Finally, the sections were dehydrated with gradient alcohol, transparent with xylene, and mounted with neutral gum.

### Statistical Analysis

Statistical analysis was performed using the SPSS program 23.0. Data were expressed as mean ± standard deviation (SD). Statistical comparisons were performed using Student’s *t*-test and one-way analysis. Values of *p* < 0.05 indicated statistically significant differences.

## Results

### Identification of CTFs Components

The components of CTFs were analyzed by UHPLC MS/MS. A total of 68 components were identified. Using the TCSMP database based on ADME parameters, 23 compounds met the screening criteria (OB ≥ 30% and DL ≥ 0.18). The details of 23 compounds are shown in Table 1.

**TABLE 1:** The active ingredients with anti-lung cancer activity from CTFs.

Metabolites	Q1:Mass to charge ratio (m/z)	RT (min)	MolID	CID	MW (g/mol)	MF	Structure
(-)-Epigallocatechin gallate	457.017	4.38	MOL006821	65,064	458.4	C22H18O11	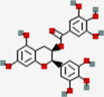
Acacetin	285.3	13.14	MOL001689	5,280,442	284.28	C16H12O5	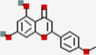
Astilbin	448.948	6.25	MOL004575	119,258	450.43	C21H22O11	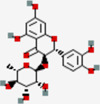
Baicalein	266.901	7.58	MOL002714	5,281,605	270.25	C15H10O5	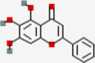
Cianidanol	290.94	3.5	MOL000492	9064	290.29	C15H14O6	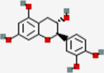
Diosmetin	301.3	10.2	MOL002881	5,281,612	300.28	C16H12O6	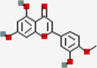
Eriodictyol	288.93	8.6	MOL005190	440,735	288.27	C15H12O6	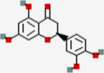
Fisetin	286.927	7.24	MOL013179	5,281,614	286.25	C15H10O6	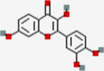
Galangin	271.2	13.54	MOL002563	5,281,616	270.25	C15H10O5	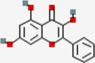
Genkwanin	284.943	13.4	MOL005573	5,281,617	284.28	C16H12O5	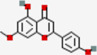
Glycitein	285.3	8.55	MOL008400	5,317,750	284.28	C16H12O5	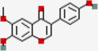
Hesperetin	303.3	10.44	MOL002341	72,281	302.3	C16H14O6	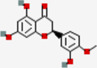
Hydroxygenkwanin	300.924	11.86	MOL005530	5,318,214	300.28	C16H12O6	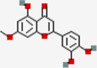
Isorhamnetin	317.085	10.46	MOL000354	5,281,654	316.28	C16H12O7	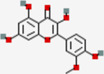
kaempferide	300.925	13.56	MOL004564	5,281,666	300.28	C16H12O6	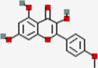
kaempferol	286.926	10.09	MOL000422	5,280,863	286.25	C15H10O6	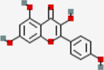
Luteolin	286.924	8.66	MOL000006	5,280,445	286.25	C15H10O6	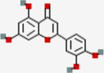
Morin	300.8	7.87	MOL000737	5,281,670	302.25	C15H10O7	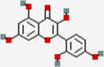
Nobiletin	403.4	13.14	MOL005828	72,344	402.43	C21H22O8	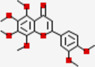
Pinocembrin	257.3	13.43	MOL002844	68,071	256.27	C15H12O4	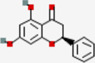
Quercetin	300.89	8.71	MOL000098	5,280,343	302.25	C15H10O7	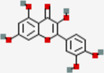
Sinensetin	372.955	12.05	MOL001803	145,659	372.4	C20H20O7	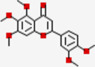
Taxifolin	304.802	6.15	MOL004576	439,533	304.27	C15H12O7	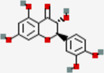

### Common-Target Network

**FIGURE 2:**
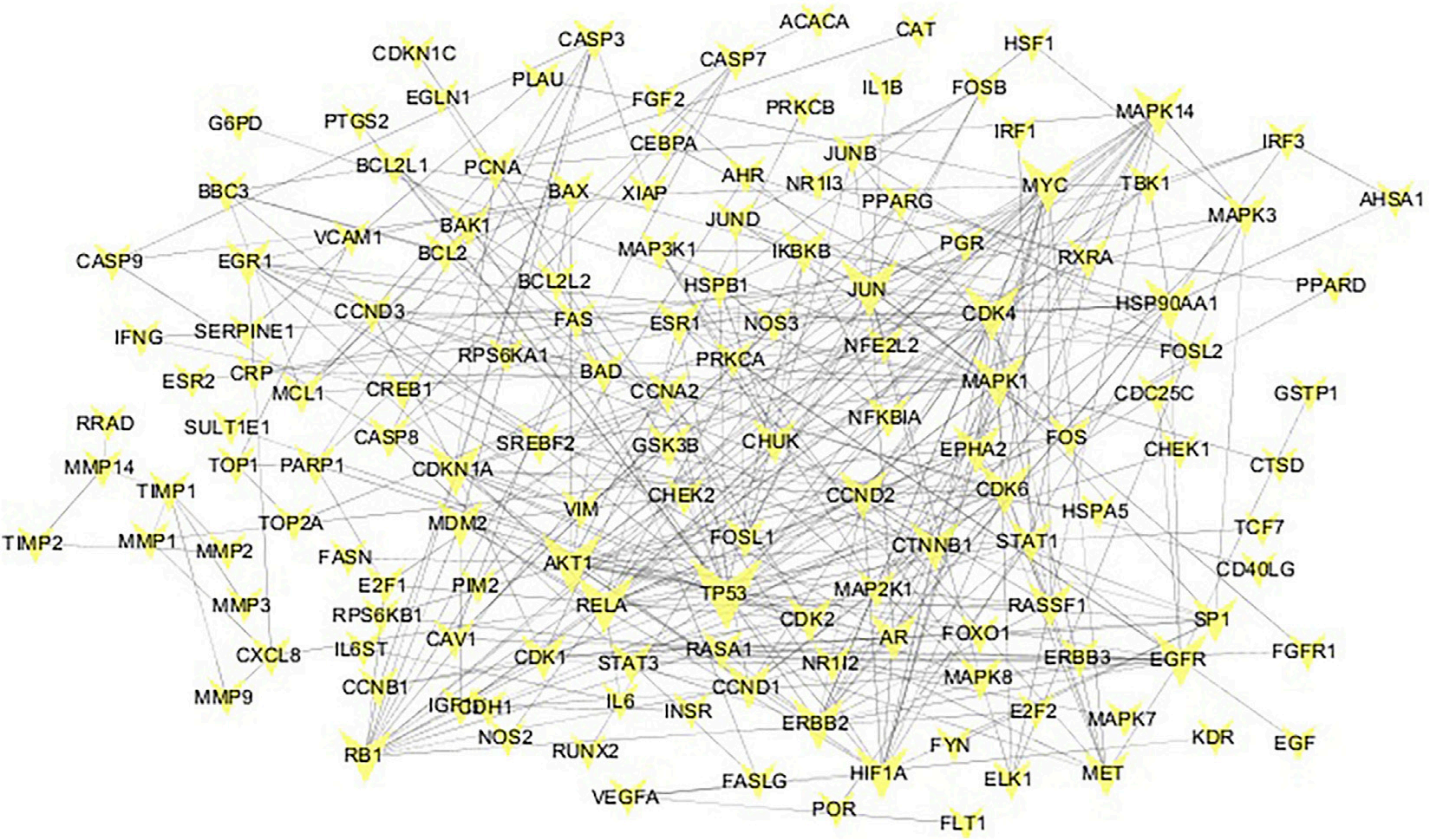
Common target network. The size of the common target node is proportional to the degree of connection. The larger the target, the greater the degree of connection.

The targets collected in the TCMSP natural product database were matched to “Homo sapiens” in the STRING database. After deduplication, 288 targets were obtained. The lung cancer-related targets collected in the OMIM and DisGeNET databases were de-duplicated and mapped to the established HINT background network. After extracting the network, compound targets were mapped to the lung cancer disease network. The largest connected pieces were extracted, and a common-target network was established (Figure 2). According to the network topology analysis, the average connectivity of the network nodes was 5.3. Thus, the 51 targets with connectivity greater than or equal to six were identified as candidate targets, which may play a pivotal role in the entire network.

### Gene Ontology and KEGG Pathway Analysis

**FIGURE 3:**
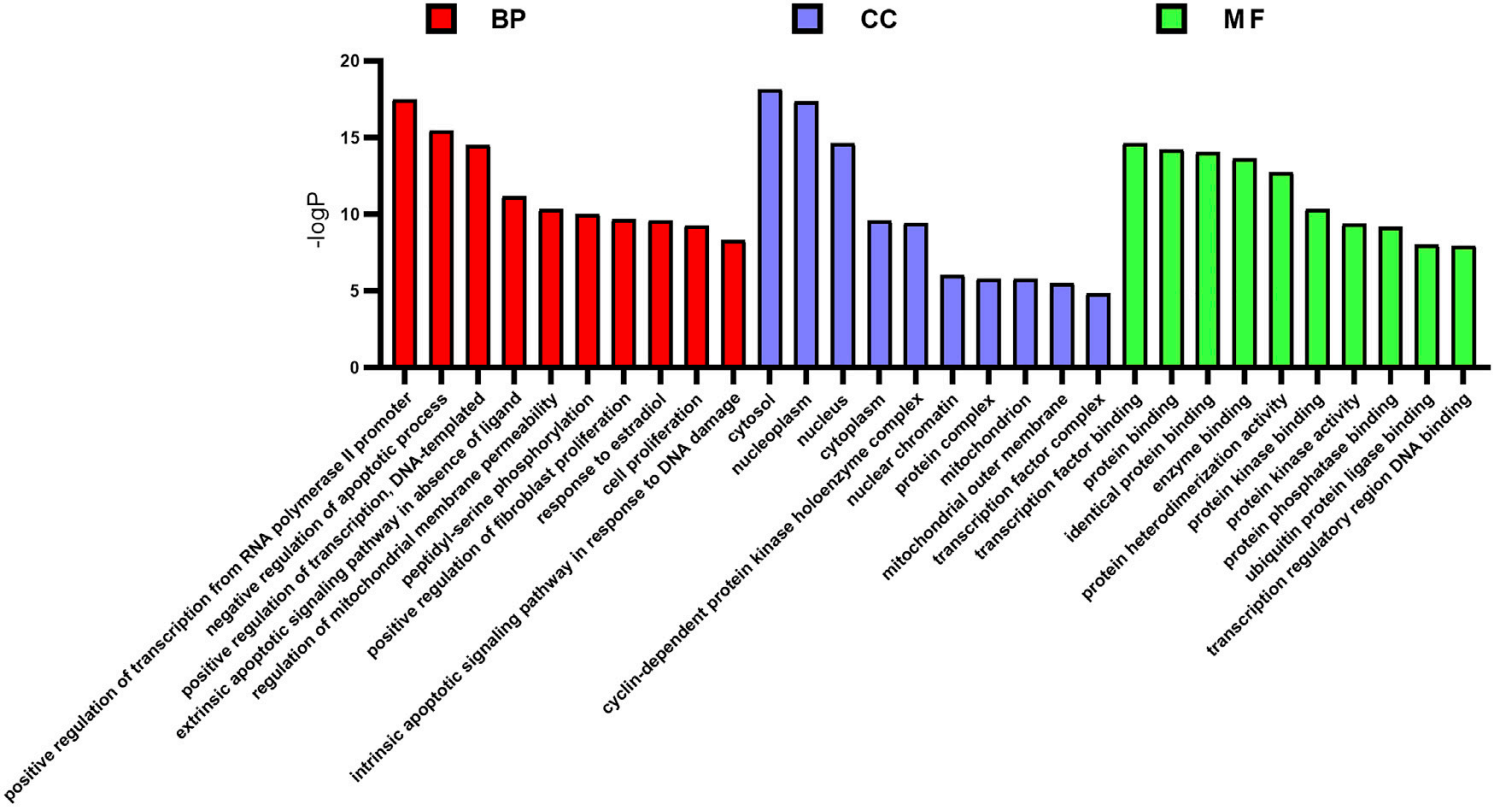
GO enrichment analysis of potential targets. The distribution of GO entries in biological process, molecular function, and cell composition (top 10 according to *p* < 0.05) are shown.

GO enrichment analyzed the functional distribution of candidate targets, and 249 GO entries were determined according to *p* < 0.01. Among them, 184 GO entries were related to biological processes, including positive regulation of transcription from RNA polymerase II promoter, negative regulation of the apoptotic process, positive regulation of transcription, DNA-templated, cell proliferation, and intrinsic apoptotic signaling pathway in response to DNA damage. There were 21 GO entries related to cell components, including cytosol, nucleoplasm, nucleus, cytoplasm, and mitochondrion; and, 44 molecular function entries, including transcription factor binding, protein binding, identical protein binding, enzyme binding, protein heterodimerization activity, etc. The top 10 (according to *p* < 0.05) in the BP, CC, and MF categories were shown in Figure 3.

**FIGURE 4:**
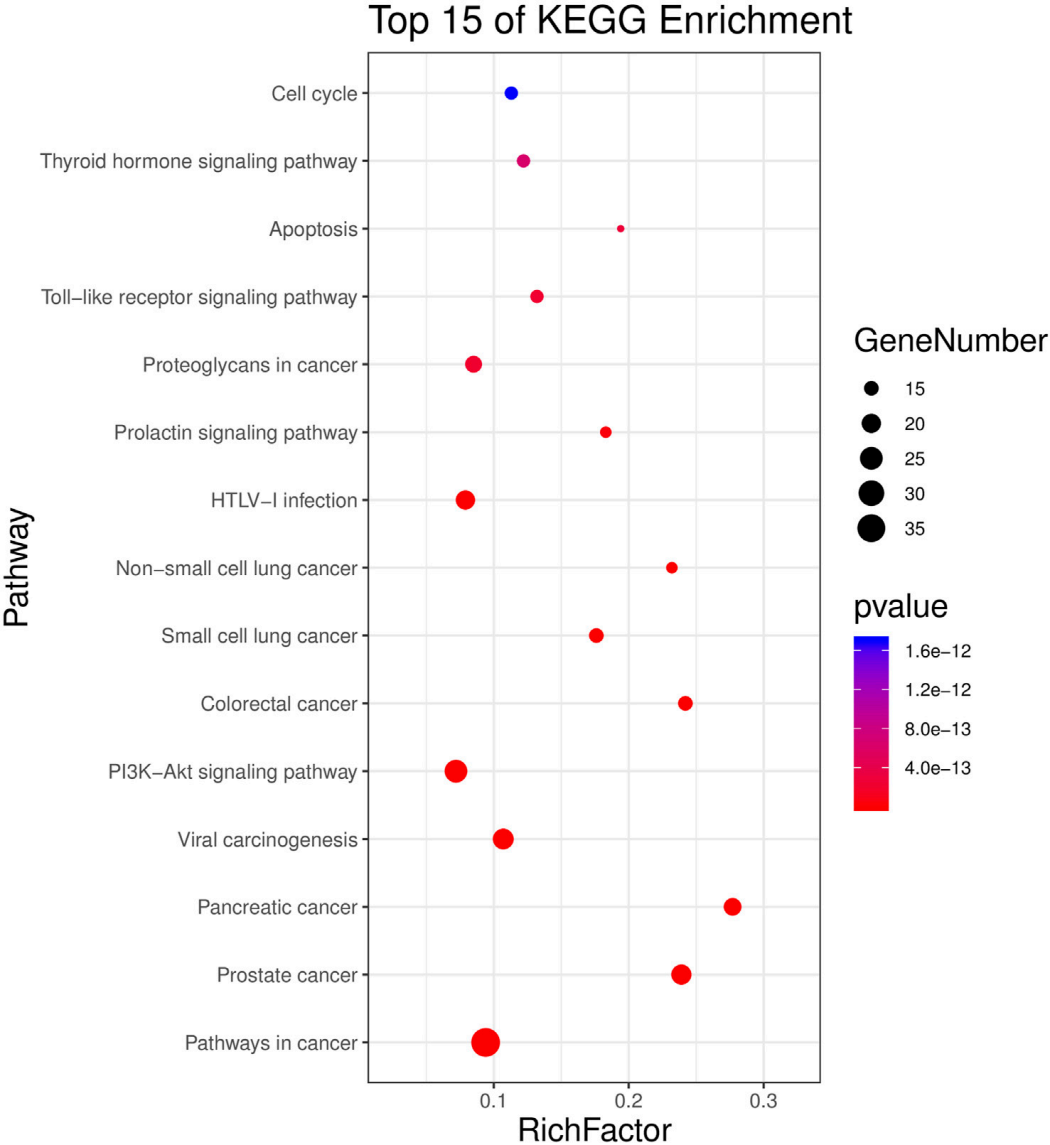
KEGG analysis for potential targets. The Y-axis represents significant KEGG pathways and the X-axis represents the rich factor. The size of the nodes shows a count of targets, and the gradient of color represents the adjusted *p*-value.

KEGG results showed that 51 targets were mainly enriched in 77 signal pathways (*p* < 0.01), primarily involving human diseases, environmental information processing, organic systems, cellular processes, such as pathways in cancer, small cell lung cancer, viral carcinogenesis, non-small cell lung cancer, PI3K-Akt signaling pathway, Toll-like receptor signaling pathway, apoptosis, cell cycle, etc. The top 15 (according to *p* < 0.05) pathways were shown in Figure 4.

### Component-Target-Pathway Network Construction

**FIGURE 5:**
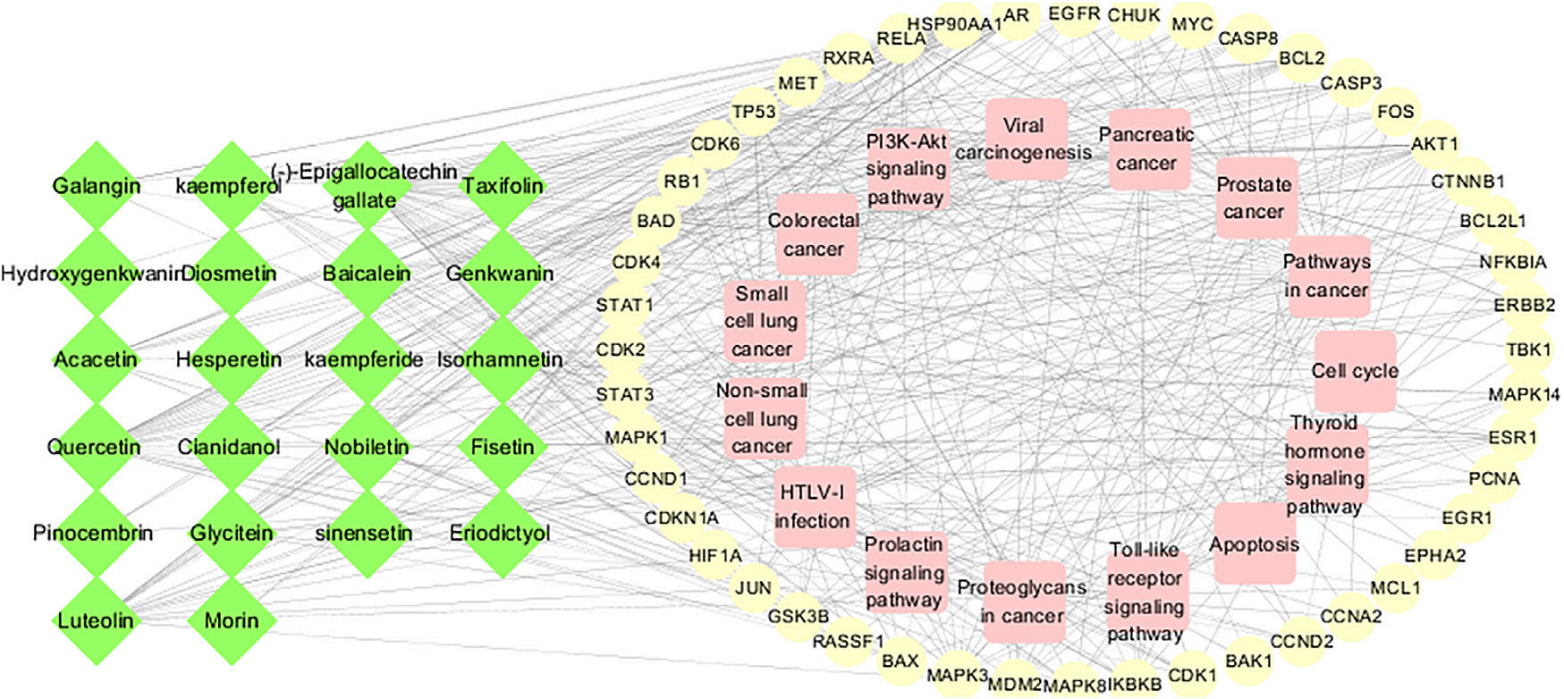
Component-target-pathway network. The green nodes of the network represent active ingredients, the yellow nodes represent potential targets, and the pink nodes represent regulatory pathways.

The 15 main pathways were obtained from the enrichment analysis corresponding to the active components and targets of CTFs, and an “active component-target-pathway” network was constructed (Figure 5). The network consisted of chemical components, protein targets, and pathways, including 85 nodes and 456 edges. Through network topology analysis, 17 protein targets with a degree (average degree of connectivity was 10.7) greater than 11 were obtained. Compared with the 51 protein targets mentioned above, these 17 protein targets were more important and may become key targets for CTFs intervention in lung cancer.

### Key Target Molecular Docking

**FIGURE 6:**
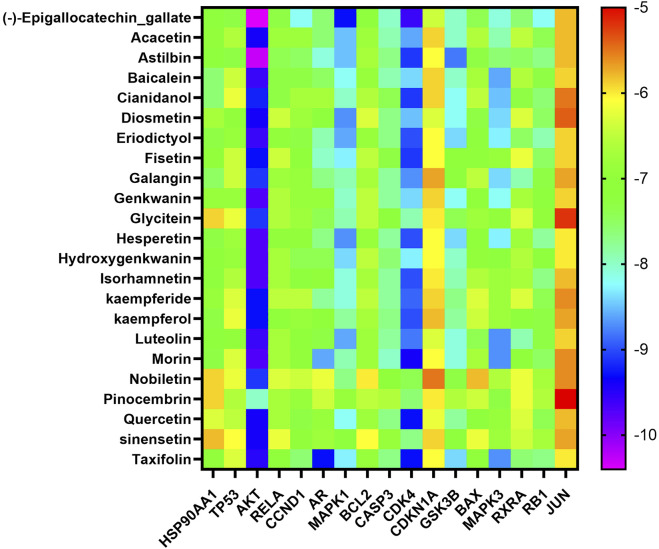
Heat map analysis of molecular docking fraction (kcal mol^−1^).

**FIGURE 7:**
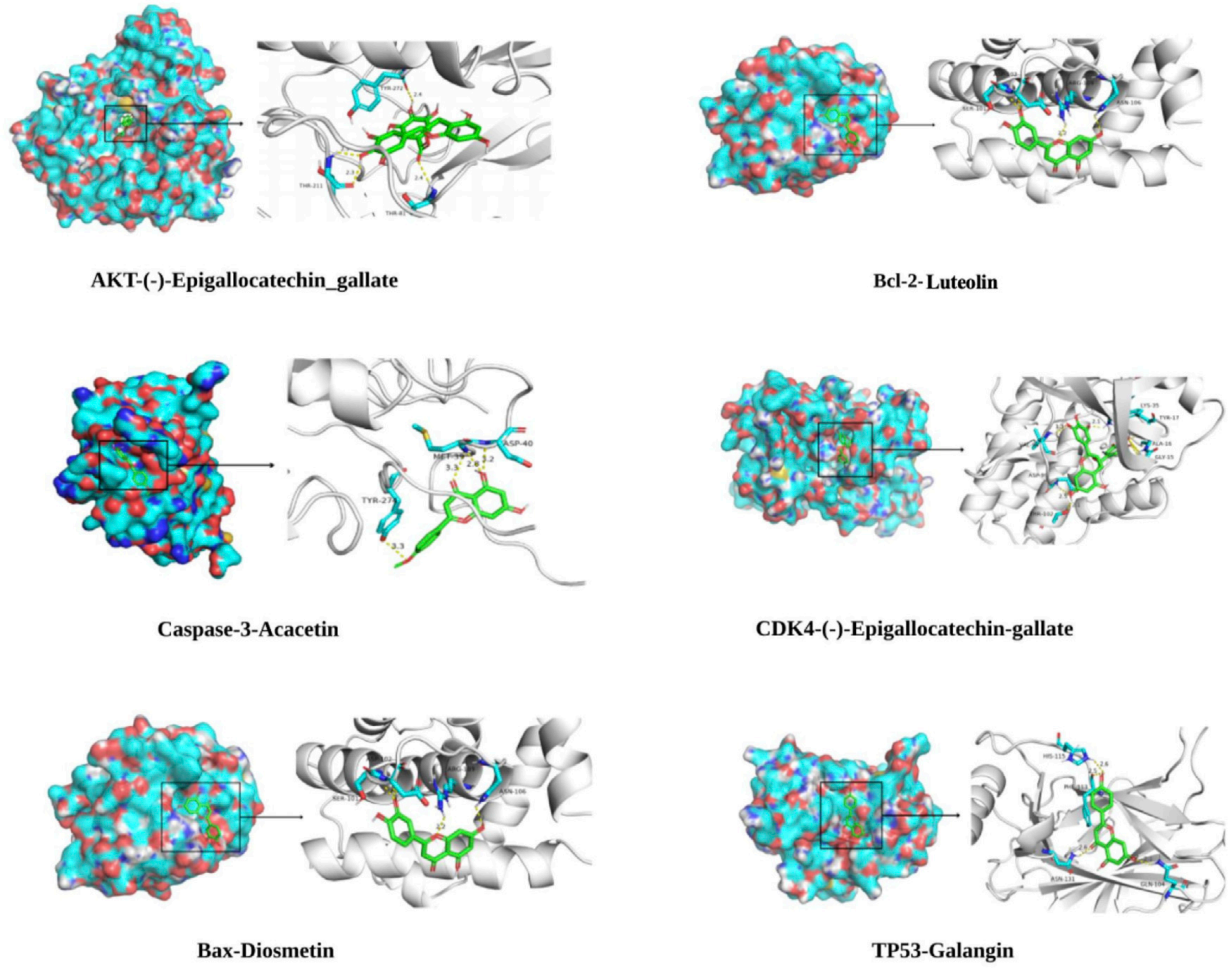
Partial molecular docking mode diagram.

In the “component-target-pathway” network, there were 17 targets with degree ≥11, and the CTFs potential active components corresponding to these targets were searched backward for molecular docking. Binding energy (affinity) < −7.0  kcal mol^−1^ indicates better binding activity (Saraswati et al., 2019), and the lower the binding energy, the better the docking effect. The results of molecular docking (Figures 6, 7) demonstrated that the active components of CTFs had good binding activity with the target proteins of lung cancer intersection. Meanwhile, these results verify the reliability of the predicted targets.

### CTFs Inhibit the Proliferation of Lung Cancer Cell

**FIGURE 8:**
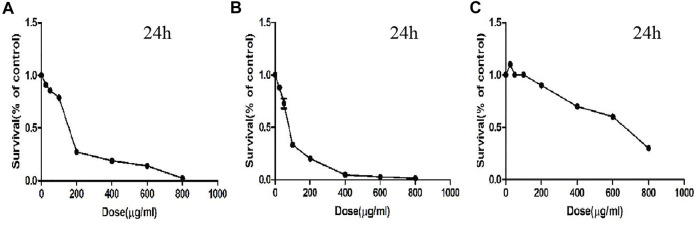
The survival effect of CTFs on cells. **(A)** A549. **(B)** H292. **(C)** BEAS-2B cells.

To detect the effect of CTFs on proliferation of lung cancer cells, CCK-8 assay was performed. After treatment with CTFs (0, 25, 50, 100, 200, 400, 600, and 800 µg/ml) for 24, 48, 72 h, the semi-inhibitory concentration (IC50) was 152.8,79.52, and 31.23 µg/ml, respectively. The IC50 of H292 was 73.58,16.10, and 13.19 µg/ml, respectively. The results showed that CTFs significantly inhibited the proliferation of A549 and H292 cells in a time- and dose-dependent manner in comparison with control group (Tables 2 and 3). However, CTFs did not suppress BEAS-2B cell viability until its concentration was over 200 µg/ml (Figure 8). Collectively, these results demonstrate that CTFs suppress the survival of lung cancer cells *in vitro*.

**TABLE 2:** Inhibitory effect of CTFs on A549 cells ( $\bar{\chi }\pm s$ , *n* = 3).

Concentrationµ(g/ml)	Inhibition rate (%)		
	24 h	48 h	72 h
Blank control group	—	—	—
Positive control group	23.79 ± 1.90*	44.14 ± 5.77*	66.94 ± 1.92*
25	9.06 ± 1.04*	16.46 ± 2.43*	49.20 ± 3.17*
50	14.01 ± 1.53*	28.80 ± 1.66*	57.66 ± 1.96*
100	21.69 ± 2.01*	55.73 ± 3.48*	72.16 ± 1.92*
200	72.59 ± 2.44*	89.83 ± 3.70*	92.26 ± 5.67*
400	81.34 ± 5.46*	96.90 ± 0.75*	98.80 ± 0.70*
600	86.10 ± 3.08*	97.66 ± 0.81*	99.60 ± 0.19*
800	98.88 ± 0.69*	98.50 ± 0.20*	99.20 ± 0.17*

*Compared with blank control group, *p* < 0.05.

**TABLE 3:** The inhibitory effect of CTFs on H292 cells ( $\bar{\chi }\pm s$ , *n* = 3).

Concentration (µg/ml)	Inhibition rate (%)		
	24 h	48 h	72 h
Blank control group	__	__	__
Positive control group	53.75 ± 4.49*	73.07 ± 2.12*	86.63 ± 1.24*
25	11.14 ± 3.14*	66.22 ± 2.42*	84.59 ± 1.42*
50	29.86 ± 1.35*	81.57 ± 0.28*	98.79 ± 0.16*
100	66.81 ± 2.15*	85.27 ± 3.02*	99.85 ± 0.09*
200	87.08 ± 1.94*	98.35 ± 0.19*	99.64 ± 0.07*
400	96.09 ± 1.31*	98.70 ± 0.36*	99.38 ± 0.11*
600	98.08 ± 0.71*	98.25 ± 0.73*	98.83 ± 0.28*
800	98.03 ± 0.38*	96.76 ± 0.12*	98.15 ± 0.03*

*Compared with blank control group, *p* < 0.05.

### CTFs Induce Apoptosis of Lung Cancer Cells

**FIGURE 9:**
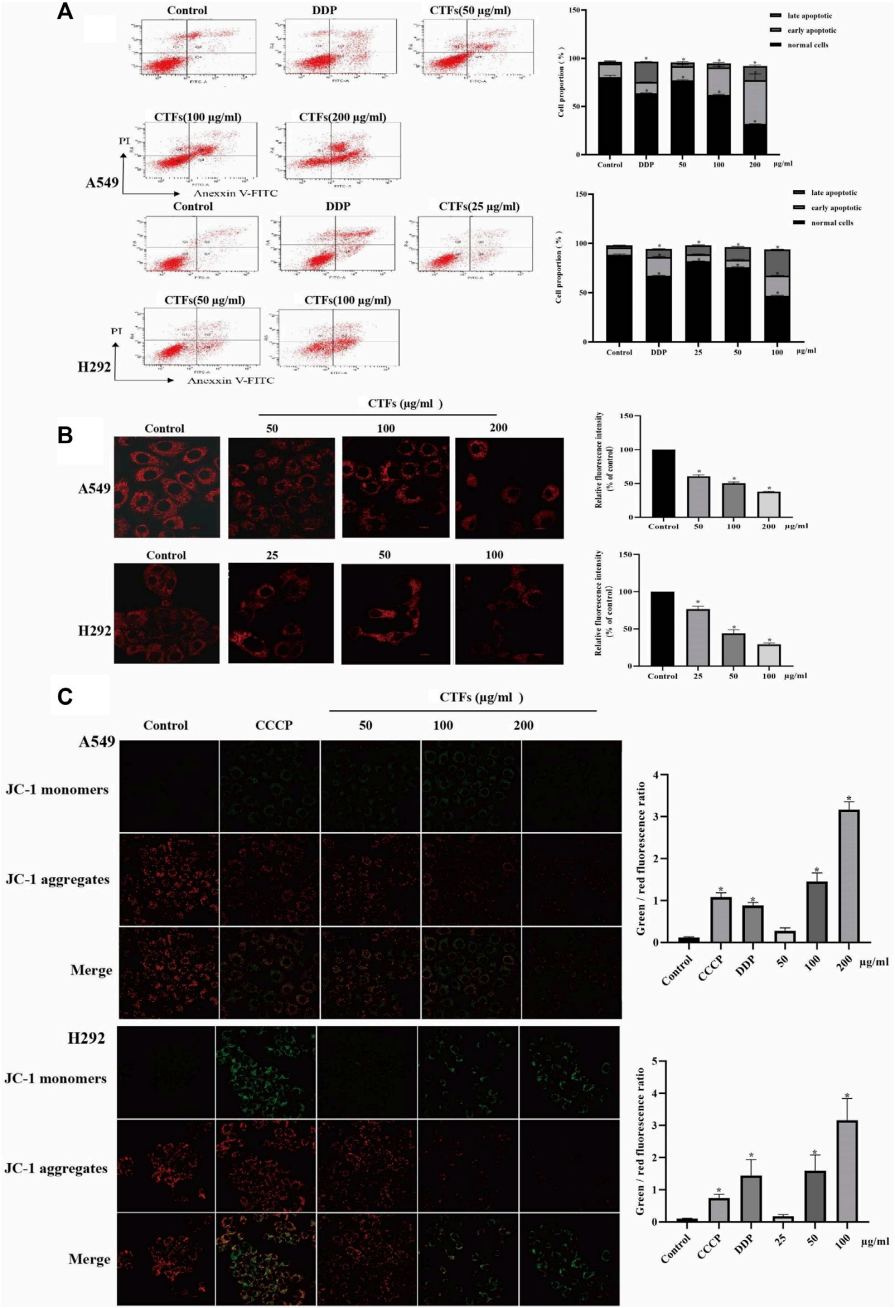
The effect of apoptosis and MMP on A549, H292 cells. **(A)** The percentage of apoptotic cells induced by CTFs treatment was detected by Annexin V/PI double-staining analysis. **(B)** Fluorescence intensity of Mito-Tracker Red CMXROS in A549, H292 cells. **(C)** The effect of CTFs on the MMP, which was determined using a JC-1 staining assay. * Compared with a blank control group, *p < 0.05*.

The flow cytometry was used to detect apoptosis. After the treatment with 50, 100, and 200 µg/ml of CTFs for 24 h, the apoptosis rate of A549 cells was 18.87 ± 1.27, 32.57 ± 0.38, 60.17 ± 5.60 vs/ 15.7 ± 2.66, respectively, compared to the control group. The apoptosis rate of H292 cells was 16.27 ± 0.55, 20.33 ± 0.15, 47.03 ± 0.64 vs/ 9.73 ± 2.16, respectively, when cells were treated with CTFs (25, 50, and 100 µg/ml). This suggested that CTFs induced apoptosis of A549 and H292 cells in a dose-dependent manner (Figure 9A). The numbers of cells in both early and late-stage apoptotic proportions were remarkably increased in cells treated with CTFs.

### Effects of CTFs on MMP of Lung Cancer Cells

Mito-tracker Red CMXRos can exhibit good photo stability to respond to changes in MMP. As shown in Figure 9B, there was a significant decrease of red fluorescence intensity in the CTFs group compared with control group (*p* < 0.01), suggesting a decrease of MMP with the presence of CTFs. To further verify this results, the MMP was also monitored by JC-1 fluorescent probe. The relative ratio of red and green fluorescence was used to evaluate the change of MMP. The results in Figure 9C showed that compared with the blank control cells, as the increase of CTFs concentration, the ratio of green fluorescence to red fluorescence increased, suggesting that CTFs can collapse the membrane potential by increasing the permeability of the mitochondrial membrane of lung cancer cells.

### CTFs Inhibit Lung Cancer Growth *in Vivo*

**FIGURE 10:**
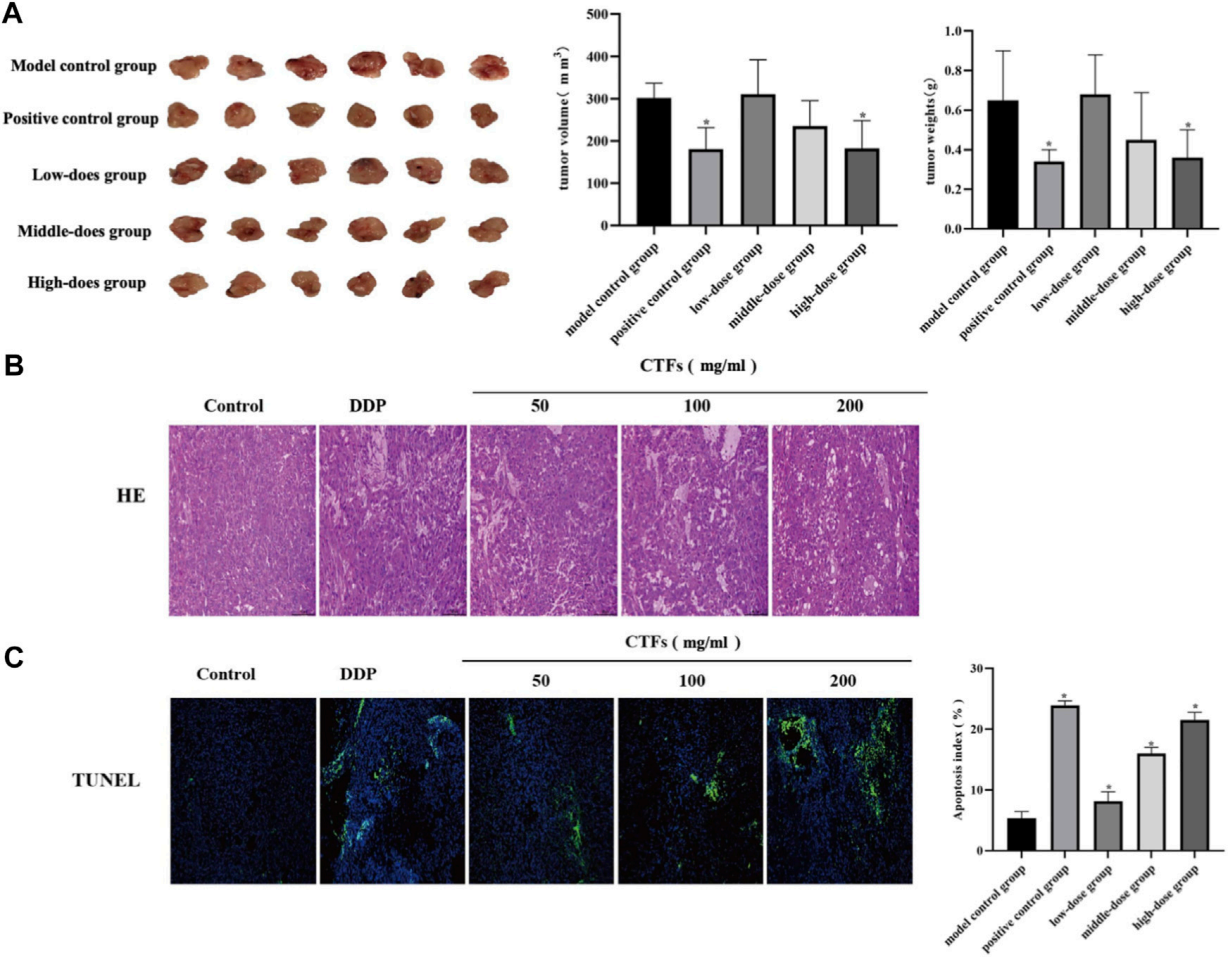
**(A)** The effect of CTFs on tumor growth in mice. **(B)** Histopathological examination of H&E staining lung tissues (×200). **(C)** The effect of CTFs on apoptosis of tumor tissue by TUNEL (×100).

To evaluate the effect of CTFs on tumor growth *in vivo*, a nude mouse model of the transplanted tumor was established. When the tumor volume reached 40 mm^3^, mice were given intervention with low, medium, and high doses of CTFs for 4 weeks. Compared with the model control group, the high-dose CTFs group significantly reduced the tumor weight and tumor volume of nude mice (0.65 ± 0.25 vs. 0.36 ± 0.14 g, 334.38 ± 26.18 vs. 204.47 ± 79.45 mm3). The tumor weight and volume of low and medium-dose group tended to decrease, but there was no statistical difference compared with the control group (Figure 10A). HE staining results showed that compared with the model group, after CTFs intervention, there were obvious damage and necrosis in the tumor tissue (Figure 10B). The results of TUNEL staining suggested that the intervention of CTFs significantly promoted the apoptosis of cells in tumor tissues (Figure 10C).

### Effects of CTFs on Apoptosis-Related Protein

**FIGURE 11:**
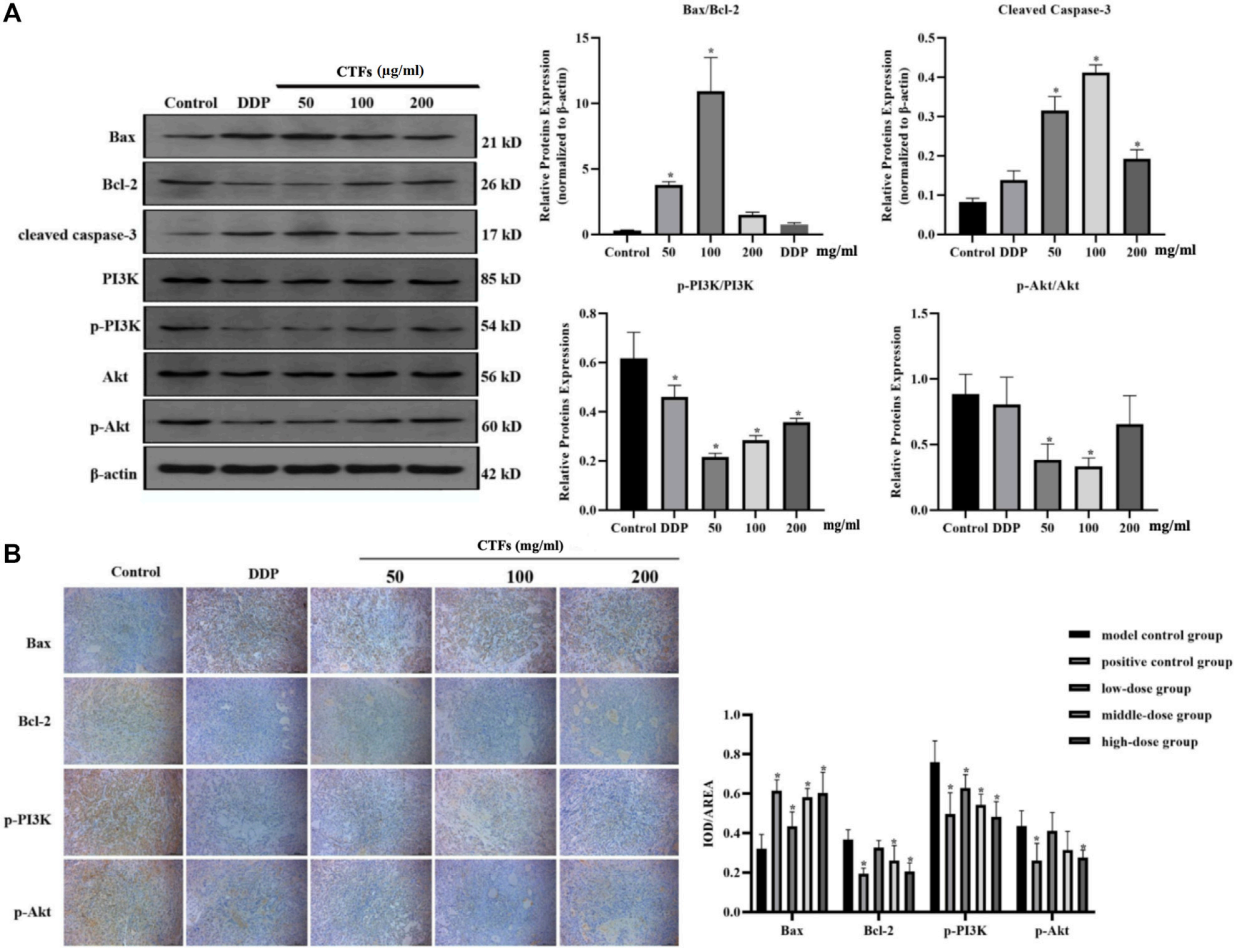
**(A)** Western blot analysis of apoptosis-related protein expression. Bax, Bcl-2, caspase-3, PI3K, p-PI3K, Akt, and p-Akt protein expression in A549 cells after CTFs treatment (50, 100, and 200 µg/ml) for 24 h. Densitometric analysis of protein expression. **(B)** The representative images and statistical graph of immunofluorescence staining for Bax, Bcl-2, PI3K, p-PI3K, Akt, and p-Akt in tumor tissue. Data were presented as mean standard deviation (SD), *n* = 6 rats per group. * Compared with blank control group, *p < 0.05*.

Western blot was used to detect changes in the expression of apoptosis-related proteins. The results were shown in Figure 11. Compared with the control cells, CTFs intervention significantly increased the ratio of Bax/Bcl-2 and the expression of Caspase-3 (*p* < 0.05). The levels of p-PI3K and p-Akt were significantly down-regulated (*p* < 0.05). However, PI3K and Akt protein expression levels did not change significantly (*p* > 0.05) in A549 cells (Figure 11A). Immunohistochemical results showed that Bax protein expression increased significantly after CTFs intervention, and Bcl-2, p-PI3K, and p-Akt protein expression decreased significantly (*p* < 0.05) *in vivo* (Figure 11B). The above results suggest that CTFs may regulate the expression of Bax, Bcl-2, and Caspase-3 to induce cell apoptosis through PI3K/Akt signaling pathway.

## Discussion

A large number of studies have shown that flavonoids extracted from plants have good anti-tumor activity against a variety of solid tumors (Tang et al., 2020; Iida et al., 2020). Jin et al. reported the anti-tumor effect of proanthocyanidins in Kunlun chrysanthemum on mouse liver cancer cell lines (H22 cells), cervical cancer cell lines (HeLa cells), and human esophageal cancer cells (Eca-109 cells) (Jing et al., 2015). The study by Parhati et al. showed that the crude extract, purified product, and total polysaccharides of *C. tinctoria* had significant inhibitory effect on the proliferation of human colon cancer cell lines (HCTI16 and Caco-2 cells) (Parhat et al., 2015). Our previous study has shown that flavonoids of *C. tinctoria* had anti-tumor activity against Eca-109 cells and could promote cell apoptosis by regulating the expression of Bcl-2 and Bax (Qiao et al., 2019). However, to date, these studies largely emphasized the anti-tumor effects and active ingredients in chrysanthemum extracts. It is still unclear how the active components act on target proteins and the mechanisms underlying the pharmacological effects of anti-tumor. Herein, we used network pharmacology and experimental verification to elaborate the pharmacological mechanisms of CTFs against NSCLC *in vitro* and *in vivo*.

In the present study, we found that CTFs treatment inhibited the proliferation of A549 and H292 cells *in vitro*. In addition, CTFs also suppressed the growth of NSCLC transplanted tumor *in vivo*. This indicated that CTFs had certain anti-NSCLC potential. Secondly, we elucidated the phytochemical composition of CTFs via UHPLC-MS/MS. Totally, 68 components were identified. The relatively high content of epigallocatechin gallate, nobiletin, quercetin, luteolin, and catechin in CTFs actually are effective ingredients in herbal medicines. Some of the components such as nobiletin, quercetin, and luteolin have been reported to possess anti-cancer activity against NSCLC (Moon et al., 2018; Dong et al., 2020; Yu et al., 2019). To explore the antitumor mechanism of CTFs, 23 components of CTFs were screened for further analyses using the TCSMP database based on analyses of ADME properties. Among of them, isorhamnetin is a natural flavonoid that has been investigated for inducing melanoma cell apoptosis via the PI3K/Akt and NF- κB pathways (Duan et al., 2020). Luteolin was reported to induce apoptotic cell death through activation of a p38/ROS/caspase cascade (Cho et al., 2015). Moreover, luteolin treatment was shown to has a strong anticancer effect via the HIF-1αand STAT3 signalling pathways, under hypoxic conditions (Fang et al., 2018). These key targets, such as PI3K, Akt, NF-κB, HIF-1α, Fos, MAPK, EGF, in these signaling pathways supported the conclusion of network pharmacology prediction. The network pharmacological analysis predicted that CTFs exerted its anti-NSCLC activity mainly via the PI3K-Akt signaling pathway and intrinsic apoptotic pathways.

The PI3K/AKT signaling pathway is one of the most important intracellular pathways in cancers, which plays an crucial role in cell proliferation, apoptosis, migration and invasion (Jiang et al., 2020). Constitutive activation of this signaling pathway is observed in NSCLC and many others (Wu et al., 2020). Studies have shown that a variety of plant polyphenols, such as nobiletin, epigallocatechin gallate, quercetin, etc. possess potential antitumor activities through directly inhibiting PI3K or blocking the PI3K/Akt signaling pathway (Goan et al., 2019; Almatroodi et al., 2020). Our results suggest that CTFs had promotive effects on apoptosis *in vitro* and *in vivo*. Further, CTFs at a certain concentration significantly down-regulated the protein expression of p-PI3K and p-Akt in A549 cells and tumor tissues. These results indicate that PI3K/Akt pathway is a possible mechanism of CTFs-induced apoptosis.

**FIGURE 12:**
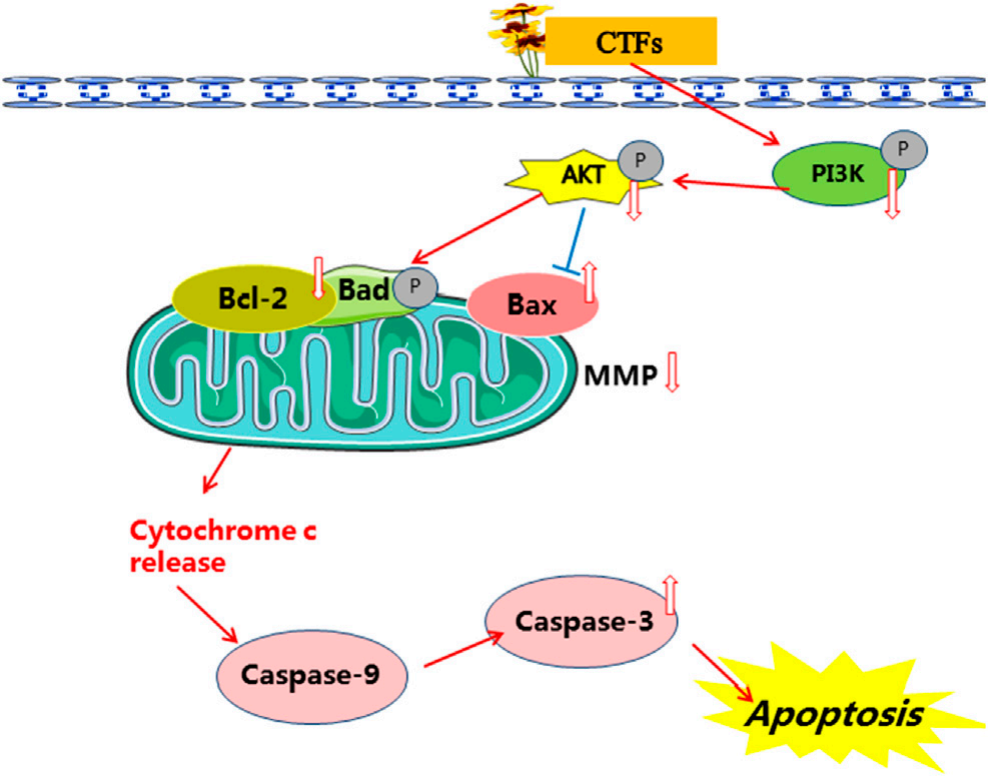
The mechanism of CTFs against lung cancer.

Apoptosis is an evolutionary conserved procedure of cell death, and activation of apoptotic pathways is an important anti-cancer strategy (Liu et al., 2019; Portugal et al., 2009). The process of apoptosis in mammalian cells mainly includes the intrinsic and extrinsic pathways. Mitochondria play important roles in the regulation of intrinsic apoptosis pathways (Green and Kroemer, 2004). Activated PI3K triggers the activations of a series of AKT downstream effectors, which initiate the expressions of the Bcl-2 family proteins. Bcl-2 family plays a key role in regulating mitochondrial function (Goan et al., 2019). During apoptosis, pro-apoptotic Bax is activated and changes the permeability of the outer membrane of mitochondria. The increase of outer membrane permeability of mitochondria will cause the decrease of MMP, resulting in the release of cytochrome C from mitochondria to cytosol, followed by caspase 9 and 3 activation (Goan et al., 2019). In agreement with these findings, our results showed that the ratio of Bax/Bcl-2 was up-regulated after CTFs treatment *in vitro* and *in vivo*. Meanwhile, CTFs treatment led to significantly reduced MMP of cells and up-regulated protein expression of cleaved caspase-3 in A549 cells. Therefore, we consider that CTFs may induce apoptosis, in part, through PI3K/Akt pathway to regulate the expression of Bcl-2 and Bax, and to active mitochondria-dependent intrinsic apoptotic pathways (Figure 12).

## Conclusion

In this study, we first demonstrated that the flavonoids, the natural compounds derived from coreopsis tinctoria Nutt, induced apoptosis and suppressed NSCLC growth. Furthermore, network pharmacology analysis predicted that the potential targets were mainly associated with PI3K-Akt signaling pathway and intrinsic apoptotic pathways. Finally, the *in vitro* and *in vivo* results supported the network pharmacology data and demonstrated that the antitumor effect of CTFs could be related to attenuation of PI3K/Akt-mediated mitochondrial depolarization and induction of caspase dependent apoptosis. Taken together, our findings suggested the antitumor components and mechanisms of CTFs, which may serve as a chemoprevention option for lung cancer. Deep analysis of anti-tumor pharmacological effects of CTFs, as well as the target and pathway acting with the active ingredients still need to be further validated.

## Data Availability

The original contributions presented in the study are included in the article/Supplementary Material, further inquiries can be directed to the corresponding author.
